# An LMI Based Criterion for Global Asymptotic Stability of Discrete-Time State-Delayed Systems with Saturation Nonlinearities

**DOI:** 10.1155/2014/761959

**Published:** 2014-10-28

**Authors:** Priyanka Kokil

**Affiliations:** Indian Institute of Information Technology, Design and Manufacturing, Kancheepuram, Chennai 600 127, India

## Abstract

A linear matrix inequality (LMI) based criterion for the global asymptotic stability of discrete-time systems with multiple state-delays employing saturation nonlinearities is presented. Numerical examples highlighting the effectiveness of the proposed criterion are given.

## 1. Introduction

When discrete-time systems are implemented in finite word length processor using fixed-point arithmetic, nonlinearities are introduced due to quantization and overflow. Such nonlinearities may result in the instability of the designed system. The global asymptotic stability of the null solution guarantees the nonexistence of limit cycles in the realized system. A number of researchers [1–20] have extensively investigated the global asymptotic stability of discrete-time systems in the presence of overflow nonlinearities.

Time delays are generally encountered in various physical, industrial, and engineering systems due to measurement and computational delays, transmission, and transport lags [21, 22]. The presence of time delays may cause instability of the designed discrete-time systems. The problem of stability analysis of discrete-time state-delayed systems has drawn the attention of many researchers [23–37].

Stability analysis of discrete-time systems in the simultaneous presence of nonlinearities and time delays in their physical models is an important problem.

This paper, therefore, deals with the problem of stability analysis of a class of discrete-time state-delayed systems in state-space realization employing saturation overflow arithmetic. The paper is organized as follows.  Section 2 introduces the system under consideration. A computationally tractable criterion for the global asymptotic stability of discrete-time state-delayed systems employing saturation overflow arithmetic is established in Section 3. It is demonstrated in Section 4 that a previously reported criterion is recovered from the presented approach as a special case. In Section 5, two examples highlighting the effectiveness of the presented approach are given.

## 2. System Description

In this section, the description of the system under consideration is given. The following notations are used throughout the paper:

 
**R** ^*p*×*q*^: set of *p* × *q* real matrices,
 
**R** ^*p*^: set of *p* × 1 real vectors,
 0: null matrix or null vector of appropriate dimensions,
 
**I**: identity matrix of appropriate dimensions,
 
**B** ^*T*^: transpose of a matrix (or vector) **B**,
 
**B** > 0: **B** is positive definite symmetric matrix,
 ||·||: any vector norm or matrix norm.

The system under consideration is given by

(1a) x(k+1)=f(y(k))=[f1(y1(k))f2(y2(k))⋯fn(yn(k))]T,

(1b) y(k)=Ax(k)+∑i=1mAdix(k−di)=[y1(k)y2(k)⋯yn(k)]T,

(1c) $$\begin{array}{c} x\left(k\right)=\varphi \left(k\right),\;\forall k=-d,-d+1,\ldots ,0, \end{array}$$

(1d) $$\begin{array}{c} d=\max\left\{d_{1},d_{2},\ldots ,d_{m}\right\}, \end{array}$$

where **x**(*k*) ∈ **R** ^*n*^ is the state vector; **A**, **A**_*d*_*i*__  (*i* = 1,2,…, *m*) ∈ **R** ^*n*×*n*^ are the known constant matrices; *d*_*i*_ (*i* = 1,2,…, *m*) is the positive integer for time delays; and **φ**(*k*) ∈ **R** ^*n*^ is the initial state value at time *k*. The function *f*_*i*_(*y*_*i*_(*k*)) representing the saturation nonlinearities given by

(2) fi(yi(k))={1,yi(k)>1yi(k),−1≤yi(k)≤1−1,yi(k)<−1,i=1,2,…,n,

is under consideration.

Let

(3) $$\begin{array}{c} \overset{-}{A}=\left[\begin{array}{ccccccccc} A & \vdots & A_{d_{1}} & \vdots & A_{d_{2}} & \vdots & \cdots & \vdots & A_{d_{m}} \end{array}\right]=\left[{\overset{-}{a}}_{ij}\right]. \end{array}$$

Define [14]

(4) $$\begin{array}{c} s_{i}=\overset{n(m+1)}{\underset{j=1}{\sum }}\left|{\overset{-}{a}}_{ij}\right|,\;i=1,2,\ldots ,n \end{array}$$

and assume that the elements of the matrix $\overset{-}{A}$ satisfy

(5a)  si>1, i=1,2,…,q,

(5b)  si≤1, i=q+1,q+2,…,n,

where *q* is an integer between 0 and *n*. Such assumption does not pose any real difficulty due to the fact that, by relabeling the states, any discrete-time system can easily be transformed into an equivalent system such that (5a) and (5b) hold.

A class of discrete-time systems can be described with (1a), (1b), (1c), (1d), and (2); it includes digital filters implemented in finite word length [1–18], digital control systems with saturation arithmetic [5], neural networks defined on hypercubes [38], and so forth.

The equilibrium state **x**_*e*_ = 0 of the system (1a), (1b), (1c), (1d), and (2) is asymptotically stable, if, for any *ε* > 0, there exists *β* > 0 such that if ||**ϕ**(*k*)|| < *β*, *k* = −*d*, −*d* + 1,…, 0, then ||**x**(*k*)|| < *ε*, for every *k* ≥ 0 and lim_*k*→*∞*_ **x**(*k*) = 0.

## 3. Main Results

In this section, a linear matrix inequality (LMI) based criterion for the global asymptotic stability of the system (1a), (1b), (1c), (1d), (2), (4), (5a), and (5b) is established.

Suppose **C** = [*c*_*ij*_] ∈ **R** ^*n*×*n*^ is a matrix characterized by

(6a) $$\begin{array}{c} \;c_{ii}\;=\overset{n}{\underset{j=1,j\neq i}{\sum }}\left(\alpha_{ij}+\beta_{ij}\right),\;i=1,2,\ldots ,q, \end{array}$$

(6b) $$\begin{array}{c} \;c_{ij}\;=\{\begin{array}{cc} \alpha_{ij}-\beta_{ij}, & \\ \;i,j=1,2,\ldots ,q\;\;\left(i\neq j\right), & \\ \frac{\alpha_{ij}-\beta_{ij}}{s_{j}}, & \\ \;i=1,2,\ldots ,q,\;\;j=q+1,q+2,\ldots ,n\;\;\left(i\neq j\right), & \end{array} \end{array}$$

(6c) αij>0,  βij>0,i=1,2,…,q, j=1,2,…,n (i≠j),

where it is implicit that, for *n* = 1, **C** corresponds to a scalar *μ* > 0.

For *n* = 3 and *q* = 2, the matrix **C** takes the form

(7) C=[α12+β12+α13+β13α12−β12α13−β13s3α21−β21α21+β21+α23+β23α23−β23s3c31c32c33],

where *α*_*ij*_ > 0 and *β*_*ij*_ > 0  *i* = 1,2, *j* = 1,2, 3 (*i* ≠ *j*).

Now, we have the following lemma.

**Lemma 1 .**

The matrix **C** = [*c*_*ij*_] ∈ **R** ^*n*×*n*^ defined by (6a), (6b), and (6c) satisfies

(8) cii≥∑j=1,j≠iq|cij|+∑j=q+1,j≠insj|cij|,i=1,2,…,q.

**Proof**

Using (6a), (6b), and (6c), we obtain

(9) cii=∑j=1,j≠in(αij+βij)=∑j=1,j≠iq(αij+βij)+∑j=q+1,j≠in(αij+βij)>∑j=1,j≠iq|αij−βij|+∑j=q+1,j≠insj|αij−βij|sj=∑j=1,j≠iq|cij|+∑j=q+1,j≠insj|cij|, i=1,2,…,q.

This completes the proof of Lemma 1.

Now, we prove our main result.

**Theorem 2 .**

The zero solution of the system described by (1a), (1b), (1c), (1d), (2), (4), (5a), and (5b) is globally asymptotically stable if there exist positive scalars *α*_*ij*_, *β*_*ij*_ *i* = 1,2,…, *q*, *j* = 1,2,…, *n* (*i* ≠ *j*) and positive definite symmetric matrices **P** ∈ **R** ^*n*×*n*^, **Q**_*i*_ ∈ **R** ^*n*×*n*^ (*i* = 1,2,…, *m*) such that the following LMI holds:

(10) Z=[P−∑i=1mQi0…0−ATC0Q1…0−Ad1TC⋮⋮⋱⋮⋮00…Qm−AdmTC−CTA−CTAd1…−CTAdm−P+C+CT]>0,

where **C** is characterized by (6a), (6b), and (6c).

**Proof**

Let

(11) $$\begin{array}{c} \hat{x}\left(k\right)={\left[\begin{array}{ccccc} x^{T}\left(k\right) & x^{T}\left(k-d_{1}\right) & x^{T}\left(k-d_{2}\right) & \cdots & x^{T}\left(k-d_{m}\right) \end{array}\right]}^{T}. \end{array}$$

In view of (1a), (1b), (1c), (1d), we have

(12) $$\begin{array}{c} \left|{\hat{x}}_{i}\left(k\right)\right|\leq 1,\;i=1,2,\ldots ,n\left(m+1\right). \end{array}$$

Using (1b), (12), and (5b), one obtains

(13) |yi(k)|=|∑j=1n(m+1)a−ijx^j(k)|≤∑j=1n(m+1)|a−ij||x^j(k)|≤∑j=1n(m+1)|a−ij|=si, i=1,2,…,n.

It follows from (5b) and (13) that

(14) $$\begin{array}{c} \left|y_{i}\left(k\right)\right|\leq 1,\;i=q+1,q+2,\ldots ,n, \end{array}$$

which, together with (2), yields

(15) fi(yi(k))=yi(k), i=q+1,q+2,…,n.

Consider a quadratic Lyapunov function [19]

(16) $$\begin{array}{c} v\left(x\left(k\right)\right)=x^{T}\left(k\right)Px\left(k\right)+\overset{m}{\underset{i=1}{\sum }}\;\overset{-1}{\underset{j=-d_{i}}{\sum }}x^{T}\left(k+j\right)Q_{i}x\left(k+j\right). \end{array}$$

Application of (16) to (1a), (1b), (1c), and (1d) gives

(17) Δv(x(k))=v(x(k+1))−v(x(k))=fT(y(k))Pf(y(k))−xT(k)[P−∑i=1mQi]x(k) −∑i=1mxT(k−di)Qix(k−di).

Now choose the quantity “*δ*” as [14]

(18) δ=2∑i=1q[yi(k)−fi(yi(k))]×[ciifi(yi(k))+∑j=1,j≠iqcijfj(yj(k))  +∑j=q+1,j≠incijsjfj(yj(k))sj]+2∑i=q+1n[yi(k)−fi(yi(k))]×[ciifi(yi(k))+∑j=1,j≠incijfj(yj(k))],

when *n* ≥ 2 and

(19) $$\begin{array}{c} \delta =2\mu \left[y_{1}\left(k\right)-f_{1}\left(y_{1}\left(k\right)\right)\right]f_{1}\left(y_{1}\left(k\right)\right), \end{array}$$

when *n* = 1.

From (13) and (15), we obtain

(20) $$\begin{array}{c} \left|f_{i}\left(y_{i}\left(k\right)\right)\right|=\left|y_{i}\left(k\right)\right|\leq s_{i},\;i=q+1,q+2,\ldots ,n. \end{array}$$

Therefore,

(21) $$\begin{array}{c} \left|\frac{f_{j}\left(y_{j}\left(k\right)\right)}{s_{j}}\right|\leq 1,\;j=q+1,q+2,\ldots ,n. \end{array}$$

Using Lemma 1 and (21), it is easy to show that the first term of (18) is nonnegative for the nonlinearities given by (2) if (8) is satisfied. In view of (15), the second term of (18) is zero. Thus, the quantity “*δ*” given by (18) is nonnegative. Equation (18) can also be expressed as

(22) δ=yT(k)Cf(y(k))+fT(y(k))CTy(k)−fT(y(k))(C+CT)f(y(k)).

Adding to and subtracting from (17), the quantity “*δ*” yields, after some rearrangement,

(23) $$\begin{array}{c} \Delta v\left(x\left(k\right)\right)=-{\tilde{x}}^{T}\left(k\right)Z\tilde{x}\left(k\right)-\delta , \end{array}$$

where

(24) $$\begin{array}{c} {\tilde{x}}^{T}\left(k\right)=\left[\begin{array}{cc} {\hat{x}}^{T}\left(k\right) & f^{T}\left(y\left(k\right)\right) \end{array}\right], \end{array}$$

and **Z** is given by (10). Therefore, if **Z** > 0, then Δ*v*(**x**(*k*)) < 0 for $\tilde{x}(k)\neq 0$. Thus, condition **Z** > 0is a sufficient condition for the global asymptotic stability of the system (1a), (1b), (1c), (1d), (2), (4), (5a), (5b), and Δ*v*(**x**(*k*)) = 0 only when $\tilde{x}(k)=0$. This completes the proof of Theorem 2.

**Remark 3 .**

The matrix inequality (10) is linear in the unknown parameters *α*_*ij*_, *β*_*ij*_ (*i* = 1,2,…, *q*, *j* = 1,2,…, *n* (*i* ≠ *j*)), **P**, and **Q**_*i*_ (*i* = 1,2,…, *m*). Thus, it can be easily solved using MATLAB LMI toolbox [39, 40].

**Remark 4 .**

Note that condition (10) is independent of the delay. Therefore, one need not know the size of the delays to establish the global asymptotic stability of the system (1a), (1b), (1c), (1d), (2), (4), (5a) and (5b) via Theorem 2.

**Remark 5 .**

Condition (10) provides a limit cycle-free realizability condition for the system with saturation arithmetic.

**Remark 6 .**

Stability of the system can be established via Theorem 2 for one combination of the elements of the matrix $\overset{-}{A}$, that is, where the elements of first *q* rows of $\overset{-}{A}$ satisfy (5a) and those of the remaining (*n* − *q*) rows satisfy (5b). The stability results for the other possible combinations of the elements of matrix $\overset{-}{A}$ can easily be worked out.

## 4. Comparison

In this section, we will compare the main result of this paper with the result stated in [41].

**Theorem 7 (see [41]).**

The zero solution of the system described by (1a), (1b), (1c), (1d), (2), and (4) is globally asymptotically stable if there exist *n* × *n* positive definite symmetric matrices **P** = [*p*_*ij*_] and **Q**_*i*_ (*i* = 1,2,…, *m*) such that

(25) $$\begin{array}{c} \left[\begin{array}{ccccc} P-\overset{m}{\underset{i=1}{\sum }}Q_{i} & 0 & \ldots & 0 & -A^{T}P\\ 0 & Q_{1} & \ldots & 0 & -{A_{d_{1}}}^{T}P\\ \vdots & \vdots & \ddots & \vdots & \vdots \\ 0 & 0 & \ldots & Q_{m} & -{A_{d_{m}}}^{T}P\\ -PA & -PA_{d_{1}} & \ldots & -PA_{d_{m}} & P \end{array}\right]>0, \end{array}$$

(26) $$\begin{array}{c} p_{ii}^{}\geq \overset{q}{\underset{j=1,j\neq i}{\sum }}\left|p_{ij}^{}\right|+\overset{n}{\underset{j=q+1,j\neq i}{\sum }}k_{j}\left|p_{ij}^{}\right|,\;i=1,2,\ldots ,q. \end{array}$$

**Proposition 8 .**

Theorem 2 implies Theorem 7.

**Proof**

It can be easily conceived that, with

(27) $$\begin{array}{c} C=C^{T}=P, \end{array}$$

matrix **C** reduces to a positive definite symmetric matrix **P**; as a result, (10) reduces to (25). Therefore, Theorem 7 is recovered from Theorem 2 as a special case.

**Remark 9 .**

The present work may be treated as an extension of [41]. Moreover, the present approach leads to generalized and improved result over the result appearing in [41].

## 5. Numerical Examples

In this section, two numerical examples are given to demonstrate the usefulness of the present result.

**Example 1 .**

Consider a second-order system (1a), (1b), (1c), (1d), (2), (4), (5a), and (5b) with

(28) $$\begin{array}{c} A=\left[\begin{array}{cc} 1.7 & -2.5\\ 0.3 & 0.1 \end{array}\right],\;\;A_{d_{1}}=\left[\begin{array}{cc} 0 & 0.001\\ 0.001 & 0 \end{array}\right]. \end{array}$$

Here, *s*_1_ = 4.201 > 1, *s*_2_ = 0.401 < 1, *m* = 1, and *q* = 1. Using MATLAB LMI toolbox [39, 40], it can be verified that Theorem 7 does not provide any feasible solution for this example.

We now apply Theorem 2 in the example under consideration. To check the feasibility of (10), we choose the matrix **C** in the following form:

(29) $$\begin{array}{c} C=\left[\begin{array}{cc} \alpha_{12}+\beta_{12} & \frac{\alpha_{12}-\beta_{12}}{s_{2}}\\ c_{21} & c_{22} \end{array}\right], \end{array}$$

where *α*_12_ > 0 and *β*_12_ > 0. With the help of MATLAB LMI toolbox [39, 40], it turns out that (10) yields the following solutions for the present system:

(30) $$\begin{array}{c} P=\left[\begin{array}{cc} 3.7407 & -9.7872\\ -9.7872 & 30.3330 \end{array}\right],\;\;Q_{1}=\left[\begin{array}{cc} 0.1201 & -0.3006\\ -0.3006 & 1.0939 \end{array}\right],\\ C=\left[\begin{array}{cc} 3.5890 & -8.6693\\ -9.3390 & 27.3297 \end{array}\right],\\ \left(\alpha_{12}=0.0563,\;\;\beta_{12}=3.5327\right). \end{array}$$

**Figure 1:**
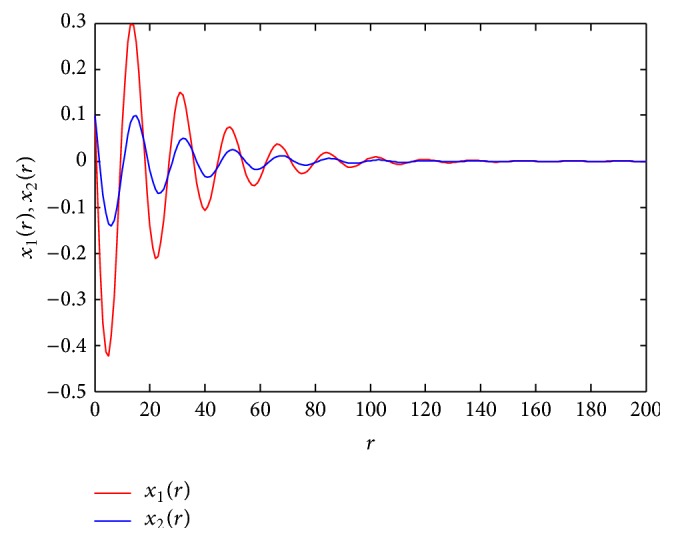
Trajectory for the state variables.

Therefore, Theorem 2 affirms the global asymptotic stability of the present system.  Figure 1 shows the trajectory of the state variable for the present example with

(31) $$\begin{array}{c} x\left(0\right)=\left[\begin{array}{c} 0.1\\ 0.1 \end{array}\right],\;\;x\left(-1\right)=\left[\begin{array}{c} 0.01\\ 0.01 \end{array}\right]. \end{array}$$

The global asymptotic stability of the system under consideration (via Theorem 2) has also been verified for a number of randomly generated initial conditions with the help of trajectories traces of the system.

**Example 2 .**

Consider a system described by (1a), (1b), (1c), (1d), (2), (4), (5a), and (5b) with

(32) $$\begin{array}{c} A=\left[\begin{array}{cc} 0.25 & -2.5\\ 0.3 & 0.1 \end{array}\right],\;\;A_{d_{1}}=\left[\begin{array}{cc} 0 & 0.001\\ 0.001 & 0 \end{array}\right],\\ A_{d_{2}}=\left[\begin{array}{cc} 0 & 0.001\\ 0.001 & 0 \end{array}\right]. \end{array}$$

Here, *s*_1_ = 2.752 > 1, *s*_2_ = 0.402 < 1, *q* = 1, and *m* = 2. Using MATLAB LMI toolbox [39, 40], it can be verified that (10) leads to the following feasible solutions:

(33) $$\begin{array}{c} P=\left[\begin{array}{cc} 46.2533 & -10.7064\\ -10.7064 & 364.9293 \end{array}\right],\\ Q_{1}=Q_{2}=\left[\begin{array}{cc} 4.6440 & -0.6299\\ -0.6299 & 25.7668 \end{array}\right],\\ C=\left[\begin{array}{cc} 47.5470 & -12.7868\\ -8.9295 & 316.4069 \end{array}\right],\\ \left(\alpha_{12}=21.2034,\;\;\beta_{12}=26.3436\right). \end{array}$$

Therefore, for this example, Theorem 2 succeeds to determine the global asymptotic stability of the system. However, (25) becomes infeasible and, consequently, Theorem 7 fails to ensure the global asymptotic stability of the present example.

## 6. Conclusions

An LMI-based sufficient condition (Theorem 2) for the global asymptotic stability of discrete-time systems with multiple state-delays employing saturation nonlinearities has been established. It is shown that Theorem 2 is less stringent than Theorem 7. Two numerical examples highlighting the usefulness of the presented result have been discussed.

The potential extensions of the proposed idea to the problems of stability of linear discrete-time systems with interval-like time-varying delay in the state [42, 43], stability of fixed-point state-space digital filters with saturation arithmetic [44], robust stability of discrete-time state-delayed systems using generalized overflow nonlinearities [19], stability of linear systems with input saturation and asymmetric constraints on the control increment or rate [45], and stability of linear two-dimensional systems with multidelays and input saturation [46], to other situations such as [47, 48], appear to be appealing problems for future investigation.
